# Estimates of microbial community stability using relative invader growth rates are robust across levels of invader species richness

**DOI:** 10.1093/ismeco/ycaf040

**Published:** 2025-03-02

**Authors:** Meaghan Castledine, Daniel Padfield, Angus Buckling

**Affiliations:** College of Life and Environmental Sciences, Environment and Sustainability Institute, University of Exeter, Penryn, Cornwall, TR10 9EZ, United Kingdom; College of Life and Environmental Sciences, Environment and Sustainability Institute, University of Exeter, Penryn, Cornwall, TR10 9EZ, United Kingdom; College of Life and Environmental Sciences, Environment and Sustainability Institute, University of Exeter, Penryn, Cornwall, TR10 9EZ, United Kingdom

**Keywords:** community stability, species coexistence, microbial community ecology, invasion criterion, relative invader growth rate

## Abstract

A key feature of natural communities is that the species within them stably coexist. A common metric used to test community stability is the ability of each species to invade from rare. A potential issue with this measurement is that single species are invaded from rare, while in natural communities, multiple species would likely decline simultaneously following perturbations. This is especially common in microbes which can be rapidly disturbed by environmental stressors. If species coexistence is dependent on indirect interactions among community members, multiple species declining may result in community instability. As such, invading a single species into a community may overestimate the stability of a community when multiple species decline. Here, we compare estimates of community stability in a five species microbial community to experimental results in which multiple species are simultaneously invaded. Our results showed that single species invasions were qualitatively predictive of whole community stability when multiple species are invaded simultaneously. However, quantitative values of relative invader growth rate were less comparable, being non-significantly different in most comparisons in three out of five species. This was emphasized by the lack of correlation between exact values of growth rates under single or multi-species invasion. This work provides experimental support for the robustness of using invasion growth rate of single species to infer qualitative estimates of community stability.

## Introduction

A key feature of many natural communities is that the species within them stably coexist [1]. Stable coexistence can arise via multiple mechanisms such as differential resource use in space and time [2–7], higher-order interactions [8], density-dependent predation [9–11] and mutualisms [12]. In theory, stable coexistence can be measured by a species’ ability to recover from rare following perturbation [1, 13–16]. Assuming species have niches distinct enough for coexistence, a rare species should display negative frequency dependent growth rates while species at higher density (residents), are limited by intraspecific competition [1, 13, 14]. The invasion criterion, which tracks the relative growth rate of rare populations compared to residents, has become a widely-used metric in community ecology [1].

A potential problem with the invasion criterion is that perturbations in nature are likely to affect more than one species simultaneously resulting in more than one species decreasing while others may be unaffected [17, 18]. If coexistence arises solely to differential resource use, the number of species simultaneously driven to low frequencies is unlikely to affect coexistence as each species is limited by intraspecific competition [14]. However, if other coexistence mechanisms are important then perturbations impacting multiple species may alter community stability. For example, if mutual invasibility relies on higher-order or intransitive interactions among resident species, multiple species becoming rare may result in a resident species (now free from interactions that limited its impact on other species) competitively excluding an invader [3, 8]. Similarly, if invasion is dependent on facilitation by another resident species, both species decreasing to low densities may result in loss of one or both species [19, 20].

Understanding the validity of these metrics when multiple species recover from rare may be particularly important for microbial ecology where species can rapidly decline and regrow following exposure to stressors. Gut and waste-water microbiomes, for example, are broadly perturbed following antibiotic applications which do not affect all species equally [18]. Soil communities are disturbed following temperature and moisture fluctuations which will increase with global warming [21]. Populations can recover when conditions become favorable again and whether recovery is successful depends on interactions between co-invaders and residents that may have increased in density [17]. In microbial ecology, stable coexistence of community members is characterized by invading a focal species at lower density than the resident species and tracking the growth rate of the invader relative to the other species present [5, 22–24]. If a community is “stable” or displays “permeance”, the invader should have a relative growth rate above 1, indicating negative frequency dependence [25]. This metric is broadly comparable to the invasion criterion used in wider ecology, although resident species are assumed to be at equilibrium which is rarely the case in microbial experiments [1], especially in batch culture.

**Figure 1:**
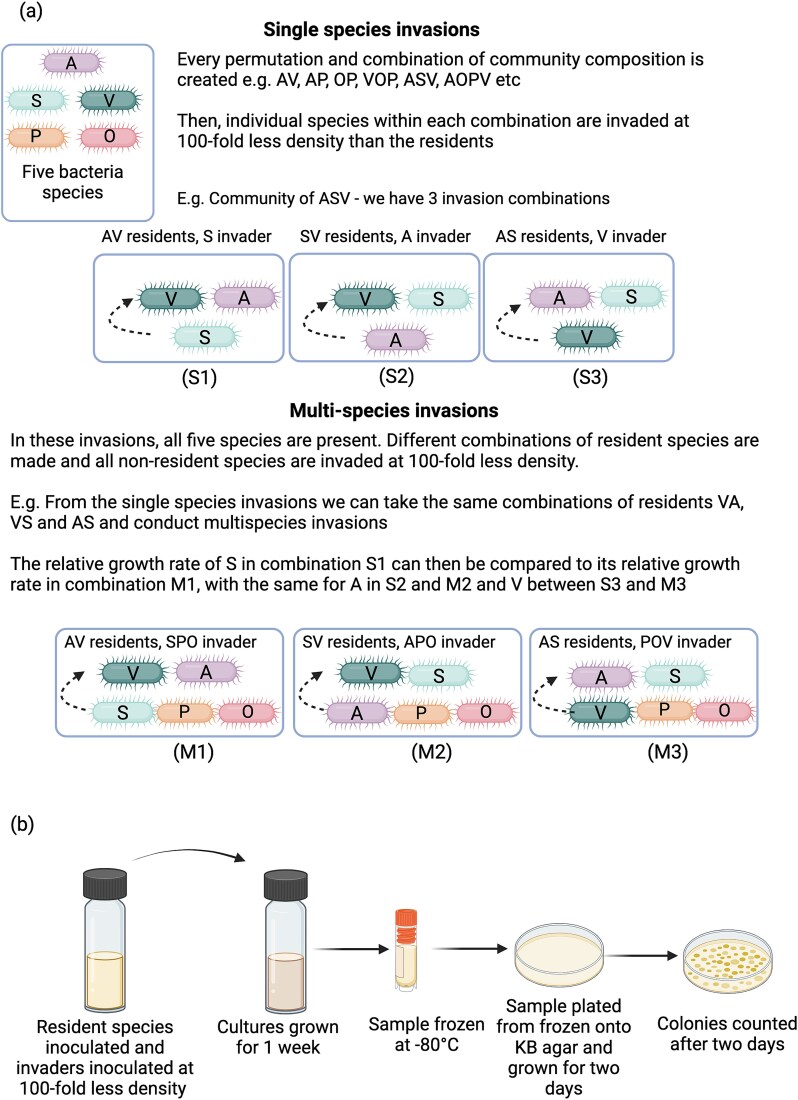
(A) Species combinations for single invasion and co-invader assays. In previous work, single species are invaded into all possible combinations of resident species from one to four resident species richness. In this study, the same combinations of residents are used with any of the five species that is not a resident being an invading species. For example, when *Achromobacter* and *Ochrobactrum* are residents, *Stenotrophomonas*, *Variovorax*, and *Pseudomonas* are invaders. As such, the relative invader growth rate of each of these invaders can be compared to when they are invading as single invaders into communities of *Achromobacter* and *Ochrobactrum*. (B) The experimental set-up is shown with species being inoculated simultaneously into liquid culture which is incubated for 1 week. A sample is frozen and plated onto KB agar from which colonies are counted to estimate species growth rates [33].

Here, we use microbial communities to address whether estimates of invasion success from single species invasions predict coexistence compared to scenarios in which multiple species invade from rare into the same resident community. Our approach specifically tests measures of community stability conducted with microbial communities in which a focal invaders growth rate is measured relative to community members. We use a five-species microbial community consisting of soil bacteria which has been shown previously to be stable [26, 27]. Interactions among the species are primarily competitive with each species inhibiting the growth of the others. *Variovorax* sp. is the only exception which derives a growth benefit from the presence of *Pseudomonas* sp., *Ochrobactrum* sp., and *Achromobacter* sp. but not *Stenotrophomonas* sp. (see Fig. 3B in 27). We previously used estimates of single invader growth rates to predict the community’s stability by decomposing the community into all two to five species combinations [27]. In a new set of experiments, we assess the ability of these metrics to predict the stability of the five-species community when multiple species are invaded from rare. We make direct comparisons between assays in which the resident species are the same. For example, in a single species invasion, *Variovorax* was invaded into a community of two species such as *Achromobacter* and *Pseudomonas*. In the multi-species invasion, we can invade *Variovorax* into the same combination of resident species, with *Stenotrophomonas* and *Ochrobactrum* present as co-invaders (Fig. 1A). We can then directly compare the relative invader fitness of *Variovorax* into a community of *Achromobacter* and *Pseudomonas* with and without co-invaders present. As many of the interspecies interactions are inhibitory, we can also predict that co-invaders would decrease the relative invader fitness of a focal species; however, whether this leads to community instability will depend on the effects of wider exploitative and indirect interactions which we previously detected [27].

## Materials and methods

Bacteria species were originally isolated from soil and include: *Achromobacter* sp. (A), *Ochrobactrum* sp. (O), *Pseudomonas* sp. (P), *Stenotrophomonas* sp. (S), and *Variovorax* sp. (V) [27]. Species can be identified by their unique colony morphology when plated on King’s medium B (KB) agar [27]. In previous work, the stability of this community was characterized by constructing every possible combination of species, from two to five species richness. Within each combination, single focal species were invaded from rare (100-fold lower than resident species) (Fig. 1). A community combination was defined as stable if the focal species’ growth rate compared to the resident species growth rates (relative growth rate) was >1. In most combinations the focal species had relative growth rate values >1 indicating this community to be highly stable (71/75 significantly >1 and all combinations with means >1) [27].

In this study, community stability was tested by maintaining total species richness at five (all species always present) and instead manipulating which species were invading from rare from two to four invader richness (Fig. 1). Single species invasions (one invader, four residents) were not conducted again, having been conducted in the aforementioned study. This resulted in a total of 70 treatments, each replicated six times (Supplementary File 1). Any species which was not a resident was instead an invading species. Isogenic populations were grown in 6 ml 1/64 TSB (tryptone soy broth diluted with demineralized H_2_O) for 2 days, shaking (180 rpm) at 28°C in 25 ml glass vials with loosened plastic lids. Cell density (colony-forming units [CFU]) of each species was normalized to 10^5^ CFU/μl (method described in [27]). The total number of invading species were inoculated at a 100-fold lower density (1.6 μl, 16 × 10^4^ CFUs total) than the sum of the resident community (160 μl, 16 × 10^6^ CFUs total). Each species was represented at equal ratios within each invading or resident community. Communities were cultured in fresh 1/64 TSB, static at 28°C. After 1 week, samples were cryogenically frozen (−80°C) in glycerol (900 μl of culture with 900 μl 50% glycerol). Samples were plated from frozen onto KB agar and incubated for 2 days at 28°C. Relative invader growth rate of the individual invading species was calculated as the ratio of estimated Malthusian parameters (*m*), *m*_focal_:*m*_community_ where *m*_community_ is the total density change of all the other populations combined including residents and co-invaders and *m*_focal_ is the density change of one of the invading species. *m* = ln(N_1_ / N_0_)/t where N_1_ is the final density, N_0_ is starting density, and *t* is the assay time (1 week) [28].

### Statistical analysis

All data were analysed using R v4.2.1 in R Studio and all plots for experimental data were made using the package “ggplot2” [29,30]. Model simplification was conducted using likelihood ratio tests and Tukey’s *post hoc* multiple-comparison tests were used to identify the most parsimonious model using the R package “emmeans” [31].

First, we assessed whether relative invader growth rate estimates under co-invasions significantly differed from 1 (indicating community stability). We performed independent one-sample t-tests on each combination of focal species by resident community combination, with the null-hypothesis being that the mean relative invader growth rate equals 1. A relative invader growth rate above 1 would indicate that the invader had a faster growth rate than the resident community, demonstrating negative frequency dependence. This resulted in 70 statistical tests and *P*-values were adjusted using the false discovery rate (fdr) method [32].

Next, we assessed whether estimates of relative invader growth rate correlated between single-species and co-invasions. The mean relative invader growth rate of each species within each combination of resident community was taken in both datasets. In a linear model, mean relative growth rate under co-invasion was analysed against interacting effects of mean relative growth rate under single invasion and species identity (which species the relative growth rate metrics referred to). Additionally, separate models for each species analysed the correlation between mean growth rates under co-invasion and single invasion using a Spearman’s rank correlation test. *P*-values were corrected for multiple testing using the fdr method. Next, comparisons were made of relative invader growth rate (co-invading and single invasion treatments) within each combination of resident species. Here, relative growth rate was analysed against interacting effects of treatment (focal invading species into different resident species combinations) and invasion type (co-invasion or single invasion).

## Results

### The community is stable in the majority of co-invasion combinations

Here, we invaded multiple species from rare simultaneously into the remainder of the residents of a five species community. In total, 56 out of 70 multi-species invasion combinations were stable with relative invader growth rate values significantly >1 (Table 1; Fig. 1). In 10 out of 14 of the failed invasions, *Stenotrophomonas* was the invading species with populations being below detectable density in at least one replicate of each combination. In contrast, *Achromobacter* accounted for 2 failed invasions, and *Pseudomonas* and *Variovorax* 1 failed invasion each. In these three species, mean relative invader growth rate was greater, but not significantly different, to 1. We previously reported that when single species were invaded from rare in all combinations of a 5 species community, 67 out of 70 invasions were successful (Table 1; Fig. 1) [27]. Of these failed invasions, only one of them (*Stenotrophomonas* into residents of *Achromobacter*, *Ochrobactrum*, and *Variovorax*) arose in both single and multi-species invasion assays. The other two failed invasions were otherwise successful in multi-species invasion. Overall, these results show estimates of community stability are robust across levels of resident and invader diversity for at least 4 of 5 species.

**Table 1:** Mean estimates of relative invader growth rate when focal species are invading into a community of different resident species as a single species or in the presence of co-invaders (species listed in the “co-invaders” column). Listed are combinations in which at least one invasion across the two invasion types failed (e.g. relative invader growth rate not significantly >1). *P*-values (corrected for multiple testing) indicate the difference of estimates to 1 with non-significant values (bold) indicating failed invasions. Letters dictate the first letter of each species name.

Focal species	Resident species	Co-invader	Single invasion		Co-invasion	
			Mean	*P*-value	Mean	*P*-value
A	O	PSV	2.064	<0.001	**1.298**	**0.058**
A	OPV	S	**1.12**	**0.124**	1.531	<0.001
A	OV	PS	1.235	0.022	**1.049**	**0.9**
A	PSV	O	**1.197**	**0.084**	1.549	0.001
P	AOS	V	1.331	<0.001	**1.14**	**0.618**
S	A	OPV	2.614	<0.001	**1.047**	**0.9**
S	AO	PV	1.849	<0.001	**0.681**	**0.409**
S	AOV	P	**1.394**	**0.053**	**1.26**	**0.468**
S	AP	OV	2.311	<0.001	**1.483**	**0.259**
S	AV	OP	2.225	0.001	**1.792**	**0.098**
S	O	APV	2.246	<0.001	**0.74**	**0.511**
S	OP	AV	2.256	<0.001	**1.46**	**0.425**
S	OV	AP	1.946	<0.001	**0.668**	**0.369**
S	P	AOV	2.738	<0.001	**1.117**	**0.85**
S	PV	AO	2.69	<0.001	**1.407**	**0.438**
V	AS	OP	1.362	0.014	**1.503**	**0.19**

**Figure 2:**
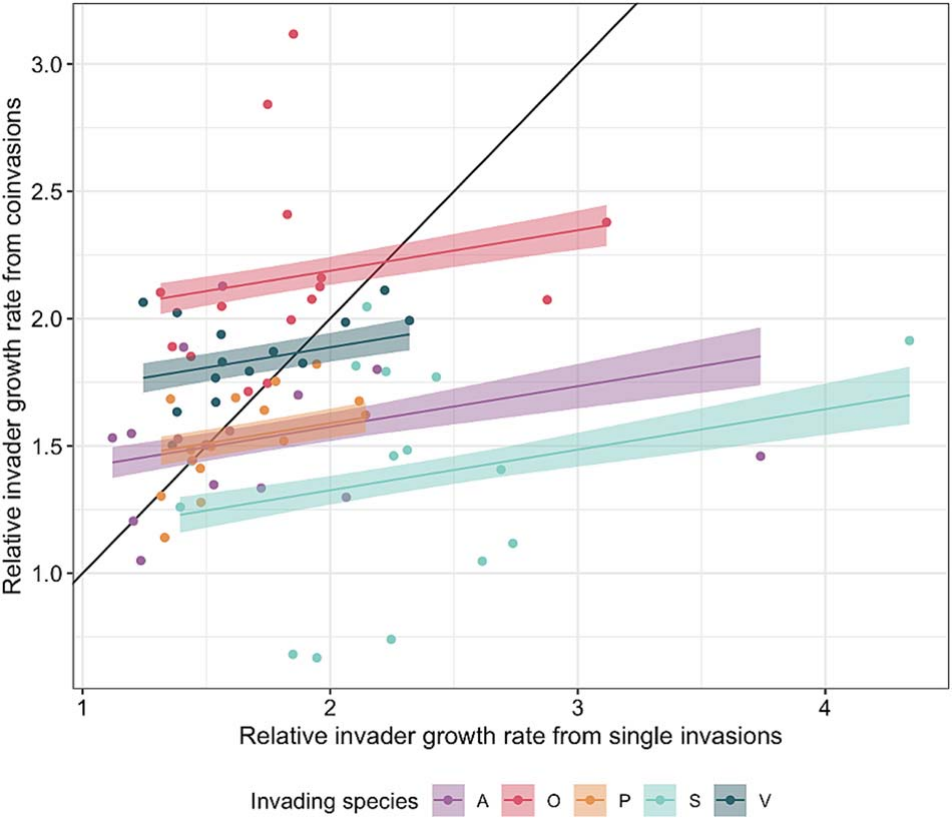
The relationship between relative invader growth rate when each species is invaded as a single species compared to the presence of co-invaders. Lines and bands represent the best fit line and 95% confidence intervals of the model. The 1–1 line indicates the slope if estimates of relative invader growth rate were directly equivalent in the presence and absence of co-invaders.

### Values of relative invader growth rate are comparable

Next, we compared mean values of relative invader growth rate between assays across each resident community combination where co-invaders are present and absent. If single species invasions perfectly predicted relative invader growth rate with co-invaders, a 1–1 relationship would be evident. Although there was a significant positive relationship between mean estimates of relative invader growth rate, the slope was only 0.156 (F_1,64_ = 4.36, *P* = .041; species-specific intercepts, F_4,64_ = 16.61, *P* < .001; interaction between relative invader growth rate for single invasions and species: F_4,60_ = 0.742, *P* = .567; Fig. 2). This means that higher relative invader growth rate estimates from single invasions do not correspond to similarly high estimates of relative invader fitness estimates from multiple invasions. However, the significant species-specific intercept indicates differences in how the two growth rate measures compare between species. For *Achromobacter* sp. (8/14), *Pseudomonas* sp. (11/14), and *Stenotrophomonas* sp. (14/14), estimates of relative invader growth rate in single invasions were higher than growth rates in co-invasion assays (Fig. 2). This suggests the presence of co-invaders decreases the relative invader growth rate of these species. In contrast, most estimates of relative invader growth rate for *Ochrobactrum* sp. (12/14) and *Variovorax* sp. (10/14) were lower than when co-invaders were present (Fig. 2). As such, for these two species, the presence of co-invaders resulted in higher estimates of relative invader growth rate. Indeed, rank correlation analyses show growth rates correlated poorly across all species: *Achromobacter* sp. (ρ = 0.007, p_fdr_ = 0.988), *Ochrobactrum* sp. (ρ = 0.433, p_fdr_ = 0.541), *Pseudomonas* sp. (ρ = 0.609, p_fdr_ = 0.118), *Stenotrophomonas* sp. (ρ = 0.222, p_fdr_ = 0.37), *Variovorax* sp. (ρ = 0.376, p_fdr_ = 0.37).

**Figure 3:**
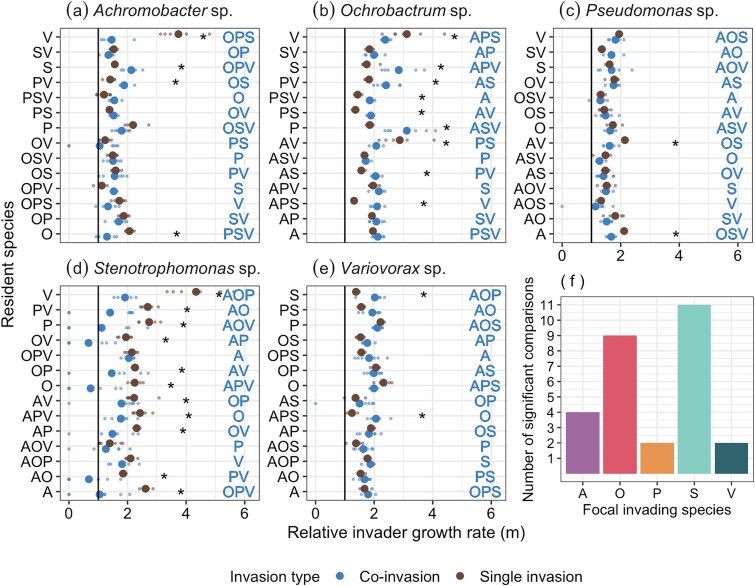
Comparisons of relative invader growth rate with focal species (A–E) invading into different combinations of resident species in the presence or absence or co-invaders. Large points indicate mean values while small points indicate values from independent treatment replicates. X = 1 is the threshold where relative invader growth rate values above this value indicates a stable community. Asterisk’s show significant differences in relative invader growth rate estimates between invasion types. Resident species combinations are a combination of the first letter of each resident species (e.g. OP = *Ochrobactrum* and *Pseudomonas*). Co-invaders, where present, are indicated in the within-figure text above the associated datapoints. Panel (F) indicates the total number of significant comparisons between co-invasion and invasion values. Species letter is the first letter from the focal species name (e.g. A = *Achromobacter*).

However, as mean estimates do not account for within treatment variation, we next compared relative invader growth rate values within combinations of resident species. Here, there was a significant interaction between treatment (focal species and resident species combination) and invasion type (presence or absence of co-invaders; F_69,700_ = 10.91, *P* < .001). Comparisons within treatments (combinations of invaders and residents) revealed no significant differences in relative invader growth rate in the presence and absence of co-invaders in 42 out of 70 comparisons. 11 out of 28 significant comparisons were accounted for by *Stenotrophomonas*, the species which had multiple failed invasions as above (Fig. 3F). 9 out of 28 significant comparisons were accounted for by *Ochrobactrum* where in 7, estimates were significantly greater with co-invaders present (Fig. 3F). *Achromobacter* accounted for 4, *Pseudomonas,* and *Variovorax* 2 each of the remaining significant comparisons (Fig. 3F). These results suggest that for most species within this community, estimates of relative invader growth rate are robust to the presence of co-invaders when accounting for within-treatment variation.

## Discussion

Relative invader growth rate is a commonly used metric for determining stable species coexistence in microbial ecology [5, 22–24]. Here, we show that single species invasions can predict community stability (i.e. the ability to reciprocally invade from rare) in most ecological scenarios in which multiple co-invaders are present. The presence of co-invaders reduced the relative growth rate of focal species resulting in a weak correlation between growth rate estimates when invading alone compared to with co-invaders. Despite this, most values of invader growth rates were above 1 and were non-significantly different between single and multispecies invasions in 3 out of 5 species. This suggests reasonably high levels of repeatability in the presence and absence of co-invaders. As such, our study suggests that the invasion criterion which typically focuses on single-species invasions can predict community stability.

*Stenotrophomonas* was predominantly affected by the presence of co-invaders and accounted for 10 out of 14 failed invasions (invader growth rates <1) while this species only had 1 failed invasion as a single invader. In some replicates, *Stenotrophomonas* was below detectable density although may not be extinct; an invader growth rate < 1 however implies that this species would be driven extinct over subsequent culturing. We do not know the mechanism by which *Stenotrophomonas* was excluded but this may be due to competitive exclusion from faster growing co-invaders and/or being of lower density in the ratio of co-invaders (lower propagule pressure compared to single invasion). However, our previous analyses of species interactions show that *Stenotrophomonas* sp. is no more affected by competition than the other species with exception of *Variovorax* sp. (see Fig. 3B in 27). Comparatively, *Ochrobactrum*’s relative growth rates were generally higher when co-invaders are present. This may suggest *Ochrobactrum* is especially affected by priority effects, with more resident species having a greater impact on invader growth rate. Both species are similarly affected by synergistic indirect effects, meaning that impacts on focal species density are stronger than predicted based on pairwise interactions, but still had species-specific responses under co-invasion (i.e. *Stenotrophomonas* sp. did worse while *Ochrobactrum* sp. growth rates were higher when co-invaders were present) [27]. Therefore, we are unable to explain these results based on our knowledge of the species’ indirect interactions. These results are in-part driven by batch culture conditions in which co-inoculation of specific species limits priority effects—we may expect stronger priority effects, especially on these two species, under sequential vs simultaneous inoculation. While a limitation of the set-up, these experiments do represent scenarios in which communities are regrowing following perturbation, albeit at different relative densities.

That co-invaders do not affect relative growth rate values for 3 out of 5 species or stability estimates for 4 of 5 species suggests that community stability is predominantly maintained by species occupying distinct niches and being limited by intra rather than interspecific competition [14, 27]. There are indirect interactions among community members which are shown to affect density but seem unlikely to affect stability estimates [27]. Although there are exploitative interactions from *Variovorax* to other community members, this interaction type appeared not to affect stability estimates as *Variovorax* was one of the 3 species where estimates were robust (12 out of 14 comparisons non-significantly different) [27]. There is selection across taxa, observed from microbes to birds and fish, to reduce competition between species and diversify into separate niches (ecological character displacement) [15, 34]. The invasion criterion has been applied to show evolution has resulted in species diversifying into separate niches with evolved populations having negative frequency dependent growth rate [35]. In such communities, the invasion criterion working with single species invasions may accurately predict community stability and the relative performance of each species when recovering from rare. It should be noted that stability via the invasion criterion should be tested following significant evolution. Although species in communities are predicted to decrease competition, species may become more antagonistic over time which may undermine community stability long-term [26].

Regarding invader growth rate estimates for mutualistic species, none of the species in this community were obligate mutualists, which may have increased the repeatability of our estimates.. Obligate mutualisms increase the risk of co-extinction following perturbation [36]. Comparing estimates of relative invader growth rates under single- and co-invasion assays when species interact mutualistically would be a next-step in understanding the broad validity of the invasion criterion in communities dominated by different interaction types.

Our community considers interactions within a trophic level (competing species) which may be representative of natural communities in contexts where the community is primarily stabilized by bottom-up interactions. However, trophic interactions, such as the presence of parasites (e.g. bacteriophages, mobile genetic elements) and predators (e.g. protists, daphnia) can also provide top-down stability on communities [37, 38]. Environmental change could have disproportionate effects on higher vs lower trophic levels, resulting in destabilizations by changing the strength of top-down pressure. For example, bacteriophages can have narrow thermal tolerances than their bacteria hosts with higher temperatures reducing infection rates [39]. Longer-term perturbations, such as climate change, could also result in communities shifting to alternative stable states with some species going extinct.

Overall, our work shows that qualitative predictions of community stability can be broadly predictive of stability under community perturbation where multiple species may recover from rare. Quantitative predictions were less reliable which appears to be a combination of species specific-traits or interactions with co-invaders and/or resident species. Future work examining community stability and the invasion criterion in more complex abiotic and biotic conditions will generate wider real-world applicability.

## Supplementary Material

treatment_combinations_ycaf040

## Data Availability

All data and R codes are available on GitHub: https://github.com/mcastledine96/Multispecies_invasions_RGR.
